# Dysbiosis of vaginal microbiota associated with persistent high-risk human papilloma virus infection

**DOI:** 10.1186/s12967-021-03201-w

**Published:** 2022-01-03

**Authors:** Ling Mei, Tao Wang, Yueyue Chen, Dongmei Wei, Yueting Zhang, Tao Cui, Jian Meng, Xiaoli Zhang, Yuqing Liu, Lisha Ding, Xiaoyu Niu

**Affiliations:** 1 Department of Gynecology, Sichuan University West China Second University Hospital, Chengdu, China; 2 Key Laboratory of Birth Defects and Related Diseases of Women and Children of Ministry of Education, Sichuan University West China Second University Hospital, Chengdu, China

**Keywords:** Vaginal microbiota, 16S rRNA sequencing, HPV persistent infection, HPV clearance

## Abstract

**Background:**

The status of vaginal microbiota in persistent high-risk human papilloma virus (HR-HPV) infection is unclear. The present work aimed to identify the vaginal microbiota of persistent HPV infection and explore the possible underlying microbiota factors.

**Methods:**

A total of 100 women were recruited in this study, of which 28 presented HR-HPV persistent infection (P group), 30 showed clearance of any subtype of HR-HPV (C group), and 42 had no history of any HR-HPV infection (NC group). The vaginal microbiota and the community structure of the three groups were compared based on the 16S rRNA sequencing of the V3–V4 region. The microbiota diversity and differential analysis were carried out to detect the potential factors associated with HR-HPV infection.

**Results:**

P and C groups showed an increase of Firmicutes and Actinobacteriota but a decrease in Proteobacteria compared to the NC group. The Chao1 index indicated that the microbial richness of the NC group was greater than C group (P < 0.05).The principal co-ordinate analysis(PCoA) revealed differences between the NC and P/C groups.The linear discriminant analysis effect size (LEfSe) method indicated that Proteobacteria phylum was significantly different in the mean relative abundance in the NC group,but the P and C groups did not show such indicative taxa. The Wilcox rank-sum test indicated that the *Bifidobacterium* (P = 0.002) and *Lactobacillus* (P = 0.005) of the C group were in a high mean relative abundance compared to the NC group.

**Conclusions:**

The persistent HR-HPV infection is associated with dysbiosis of the vaginal microbiota. Microbiome regulation with *Bifidobacteria* and *Lactobacillus* may affect the clearance of HPV.

**Supplementary Information:**

The online version contains supplementary material available at 10.1186/s12967-021-03201-w.

## Background

According to the World Health Organization (WHO) statistical data, cervical cancer is the fourth most common cancer in women. About 99% of the cases are linked to infection of high-risk human papillomavirus (HR-HPV) [1]. HPV infection is common in sexually active women and usually can be spontaneously eliminated from individuals within 6–24 months, and only a small proportion of infected women retain the HR-HPV [2]. However, epidemiological studies showed that the world prevalence of HPV infection is 11.7% [3]. Lacking a definitive treatment for the infection and the high cost of cervical cancer treatment makes HPV a major public health concern. Vaginal douching or sexual intercourse and biological factors, such as bacterial vaginosis or sexually transmitted infections (STIs) affect the vaginal microenvironment and act as cofactors in the persistent HPV infection [4–7]. Several studies explored the correlation between vaginal microbiota and HPV infection, precancerous lesions of cervix or cervical cancer. According to vaginal microbiota species composition, there were five community state types (CSTs). CST I, II, III, and V were dominated by *Lactobacillus crispatus* (*L. crispatus*), *Lactobacillus gasseri*, (*L. gasseri)*, *Lactobacillus iners* (*L. iners)*, and *Lactobacillus jessenii* (*L. jessenii)* respectively. CST IV was characterized by a high proportion of strictly anaerobic bacteria [8]. CST IV was correlated with persistent HR-HPV infection [9]. In addition, Brotman et al. [10] found that the CSTs were associated with changes in HPV status, and a small *Lactobacillus* community with high proportions of the genus *Atopobium* had the slowest rate of HPV clearance. The presence of *Anaerococcus vaginae*, *Garderella vaginalis*, and *L. iners* in the absence of *L. crispatus* were identified as the most high-risk combinations for the development of cervical cancer. Kyeong et al. [11] found that there was a marked decrease of *L. crispatus* in the CIN/cancer groups compared with that in the normal group. *Atopobium vaginae, Dialister invisus, Finegoldia magna, Gardnerella vaginalis, Prevotella buccalis* and *Prevotella timonensis* were significantly associated with the risk of CIN 2/3 or cervical cancer. So far, the majority of the studies had focused on the correlation between CSTs and HPV infection status or correlation between vaginal microbiota and the cervical lesions, while only a few studies aimed to identify the possible microbial biomarker that affects the susceptibility to HR-HPV before any cervical precancerous lesion occurred. Finding the biomarker could help clearing HPV before cervical lesions occur. Herein, we compared the composition and structure of vaginal microbiota in persistent HR-HPV infection, HR-HPV clearance, and HR-HPV-negative women based on 16S rRNA high-throughput sequencing to characterize the bacterial taxa.

## Methods

### Data and sample collection

A total of 100 women were recruited at the Gynecology Outpatient Department of West China Second Hospital in Chengdu between January 2015 and March 2021. All the women underwent the screening test for both HPV DNA detection and cytology. We use polymerase chain reaction(PCR) reverse dot blot hybridization to qualitatively detect 17 types of HR-HPV DNA, namely, 16, 18, 31, 33, 35, 39, 45, 51, 52, 53, 56, 58, 59, 66, 68, 73 and 82. The including criteria were as follows: (1) The cytological examination of the uterine cervix indicated negative intraepithelial lesions or malignancies (NILMs); (2) If HPV16 or HPV18 was positive, women were referred for colposcopy, and no cervical intraepithelial neoplasia (CIN) or cervical cancer should be detected; (3) Women with persistent infection of specific HR-HPV subtypes for ≥ 12 months comprised the P group;(4) Women with clearance of some HR-HPV subtypes after observation for 12 months comprised the C group; (5) Women without HR-HPV infection records for the recent 2 years constituted the NC group. According to the HPV subtypes, the samples from P and C groups were further divided into PC (persistent infection with subtype 16 or 18), PO (persistent infection with subtypes other than subtype 16 and 18), CC (clearance of subtype 16 or 18), and CO (clearance of subtypes other than 16 and18) subgroups. The excluding criteria were as follows: (1) Women had sexual activity in the 48 h before the visit; (2) Women used douches, vaginal medications, or had reported vaginal discharge in the past 48 h; (3) Pregnant women; (4) Women had taken any antibiotics or antimycotics, hormone replacement treatment, or oral contraceptive medication in the past 30 days; (5) Women received any antivirus treatment, such as topical interferon. After obtaining informed consent, mid-vaginal secretion samples were obtained from these women using sterile cotton swabs. For each sample, two swabs were collected in sterilized tubes on ice until genomic DNA extractions within 3 h.

### Whole genomic DNA extraction

The TIANamp Swab DNA kit (TIANGEN BIOTECH Beijing Co. Ltd) was used to extract the whole genomic DNA of vaginal bacteria species, according to the manufacturer's instructions. The swab suspensions were mixed with 20 μL proteinase K. The mixture was incubated at 56 °C for 60 min. Then, the samples were subjected to mechanical lysis by bead beating in a Fast Prep 24 machine (MPBio, USA) at 6 m/s for 40 s. The lysates were subjected to centrifugation at 12,000 rpm for 60 s to pellet the beads and filtered using the CR2 columns. DNA was purified, and the quality of total genomic DNA was evaluated by agarose gel electrophoresis (1% E-gel, Invitrogen, USA).

### DNA amplification and sequencing of 16S rRNA gene fragments

The V3-V4 region of the bacterial 16S ribosomal RNA gene was amplified by PCR (95 °C for 2 min, followed by 25 cycles at 95 °C for 30 s, 55 °C for 30 s, and 72 °C for 30 s and a final extension at 72 °C for 5 min) using primers 341F (CCTAYGGGRBGCASCAG) and 806R(GGACTACNNGGGTWTCTAAT). The 20 μL PCR reaction consisted of 10 ng template DNA, 0.8 μL of each primer (5 μM), 2μL of 2.5 mM dNTPs, 4 μL of 5X FastPfu Buffer, and 0.4μL of FastPfu polymerase. The amplicons were excised from 2% agarose gels and purified using the AxyPrep DNA Gel Extraction Kit (Axygen Biosciences, Union City, CA, USA) according to the manufacturer’s instructions. Purified PCR products were quantified using Qubit®3.0 (Life Invitrogen). The pooled DNA product was used to construct Illumina pair-end library following Illumina’s genomic DNA library preparation procedure. Then, the amplicon library was paired-end sequenced (2 × 250) on an Illumina MiSeq platform (Shanghai BIOZERON Co., Ltd), according to the standard protocols.

### Data analysis

The raw reads were deposited into the Genome Sequence Archive(GSA)database.(Accession Number: CRA005466) After merging the paired reads and chimera filtering, sequences were clustered into operational taxonomic units (OTUs) at 97% similarity using the Deblur denoising algorithm. The phylogenetic affiliation of each *16S* rRNA gene sequence was analyzed by uclust algorithm (http://www.drive5.com/usearch/manual/uclust_algo.html) against the silva (SSU138.1) 16S rRNA database using the confidence threshold of 80%.

The community structures of P, C, and NC groups were analyzed. Venn diagrams were drawn using the online tool “Draw Venn Diagram” (http://bioinformatics.psb.ugent.be/webtools/Venn) to analyze overlapped and unique OTUs during the processes. The rarefaction analysis based on Mothur v.1.21.1 was conducted to reveal the alpha diversity by Chao index. The beta diversity was analyzed using bray_curtis [12] to compare the results of the principal co-ordinates component analysis (PCA) by R-forge (Vegan 2.0 package). The biomarkers for HR-HPV infection or clearance were identified by linear discriminant analysis effect size (LEfSe) analysis [13]. Kruskal–Wallis sum-rank test was performed to examine the changes and dissimilarities among classes, followed by linear discriminant analysis (LDA) to determine the size effect of each distinctively abundant taxa. To explore the correlation between vaginal microbiota and the infection status of certain HPV subtypes, Wilcox rank-sum test was performed on the PC, PO, CC, CO, and NC groups to identify the putative biomarker for persistent HR-HPV infection or elimination of HR-HPVs.

The statistical analysis was performed using the SPSS Statistics software 17.0 (IBM, USA). Student's *t*-test, χ^2^ or Fisher's exact test, and one-way analysis of variance (ANOVA) were conducted to determine the statistical significance as appropriate. P ≤ 0.05 was considered statistically significant.

## Results

### Baseline of the P, C, and NC groups and quality assurance

The age of the cohort was 21–64 (median, 36) years old. Of these, 19 patients were in menopausal transition or postmenopause, one patient of group C had levonorgestrel intrauterine system. P group consisted of 28 cases, the C group consisted of 30 cases, and 42 cases comprised the NC group. The clinical characteristics are shown in Table 1, and the baseline was balanced.

**Table 1:** Clinical characteristics of the enrolled patients

Characteristics	P Group (N = 28)	C Group (N = 30)	NC Group (N = 42)	P
Age (years, Mean ± SD)	38.47 ± 7.37	38.86 ± 11.44	37.29 ± 7.49	0.76
Gravity(n, Mean ± SD)	1.68 ± 1.57	1.81 ± 1.60	1.48 ± 1.70	0.73
Parity (n/%)				
≤ 1	26/92.86	27/90	37/88.10	0.81
≥ 2	2/7.14	3/10	5/11.90	
Smoking history (n/%)				
No	26/92.86	29/96.67	38/90.48	
Yes	2/7.14	1/3.33	4/9.52	0.60
HR-HPV subtype (n/%)				
Negative	0/0	0/0	42/100	
HPV-16	6*/21.43	3/10	0/0	
HPV-18	4*/14.29	3/10	0/0	
Others	20^#^/71.43	24/80	0/0	

* One of the cases was infected with both HPV-16 and HPV-18

^#^One of the cases had persistent HPV-18 and HPV-58 infection

The sequences of each sample varied from 10,021 to 58,982, and the average length of the valid tags was 421.54bps. A total of 1459 OTUs were identified using a cutoff of 97% sequence similarity. Subsequently, 19 phyla, 215 genera, and 240 species were identified, and the average goods coverage index was 0.998, indicating that 99.8% of the bacteria present in our samples were likely to have been identified. An additional figure file shows this in more detail (see Additional file 1).

### *Comparison of the community structure of the P, C*,* and NC groups*

**Fig. 1:**
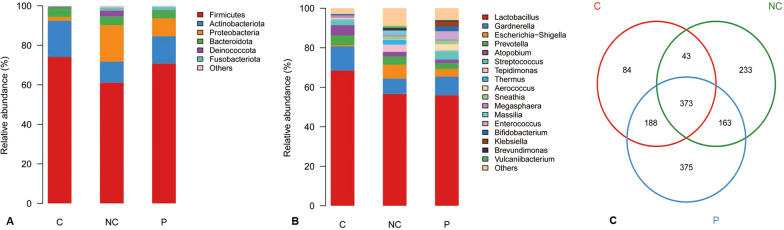
Microbial community structures of the P, C, and NC groups. **A** The microbial community bar plot of the three groups at the phyla level. **B** The microbial community bar plot of the three groups at the genus level. **C** OTU Venn diagram of the three groups. Different colors indicate various groups. The number in the overlapped area means the number of common OTUs of the groups

The microbial community analysis indicated that Firmicutes, Actinobacteriota, and Proteobacteria were the dominant three phyla in all the study groups, with relative abundances of 61.18, 10.68, and 18.47%, respectively, in the NC group, and 70.92, 13.89, and 9.08%, respectively, in the P group, and 74.26, 18.28, and 1.85%, respectively, in the C group. Samples of P and C groups were associated with an increase in Firmicutes and Actinobacteriota and decrease in Proteobacteria compared to the samples of NC group (Fig. 1A). At the genus level, the C group was associated with a bloom of *Lactobacillus* compared to the NC and P group samples. Samples of NC group were associated with an increase in *Tepidimonas* and *Escherichia-shigella* and decrease in Gardnerella compared to the samples of P and C groups (Fig. 1B).The Venn diagram revealed that there were 373 common OTUs within the samples of all the three groups. NC, P and C groups had 233, 375 and 84 unique OTUs respectively, indicating that the microbiota community of each group was varied. (Fig. 1C).

### Comparison of microbiota diversity in P, C, and NC groups

**Fig. 2:**
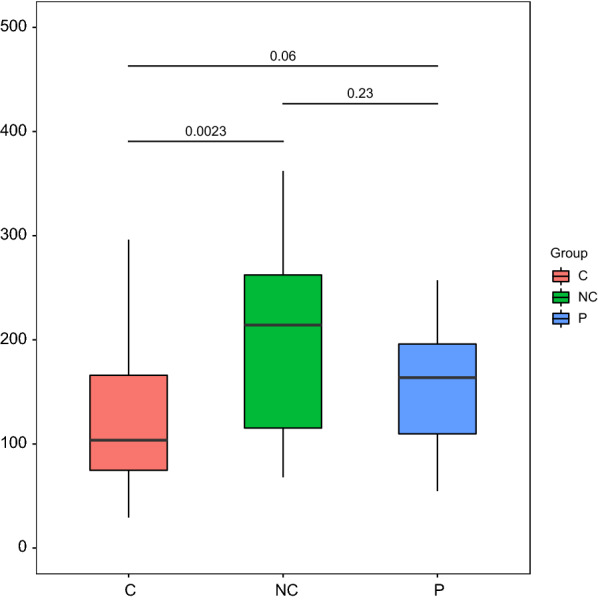
Box plot of the Chao1 index for the three groups. Each box plot represents one of the groups. The dark horizontal bar represents the median value of each group, while the boxes represent the 25th and 75th percentile values

**Fig. 3:**
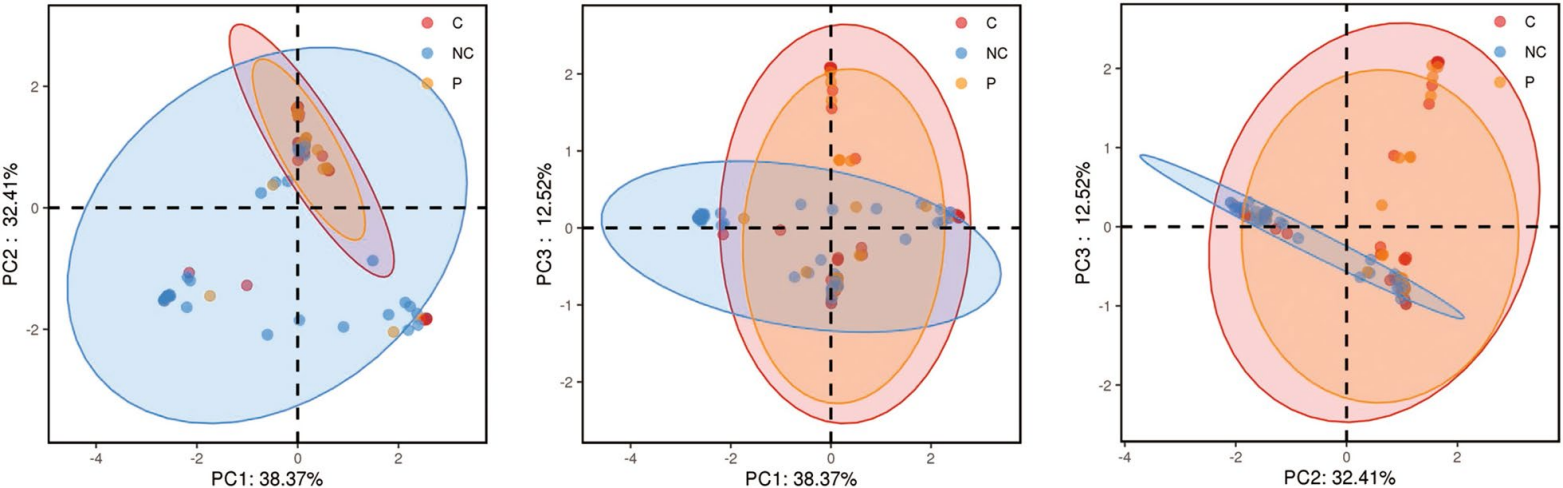
PCoA analysis of the P, C, and NC groups. NC samples are indicated in blue, P samples are in orange, and C samples are in red. The distance within the groups at the Pc1, Pc2, and Pc3 axes indicates the beta-diversity of the three groups

The Chao1 index indicated that the microbial richness of the NC group was greater than the C group with statistical difference (P < 0.05) and P group without significant statistical difference (P = 0.23). No statistical difference of microbial richness in P and C groups had been revealed. (Fig. 2).The principal co-ordinates analysis (PCoA) of bray_curtis distances was used to compare the beta diversity among the three groups. The results revealed a distinction between NC and P/C groups. The three principal component scores accounted for 38.37%, 32.41%, and 12.52% (Fig. 3).The analysis of similarity (anosim) of bray_curtis indicated a significant beta diversity difference within the three groups(P = 0.001).

### The feature taxa of P, C, and NC groups

**Fig. 4:**
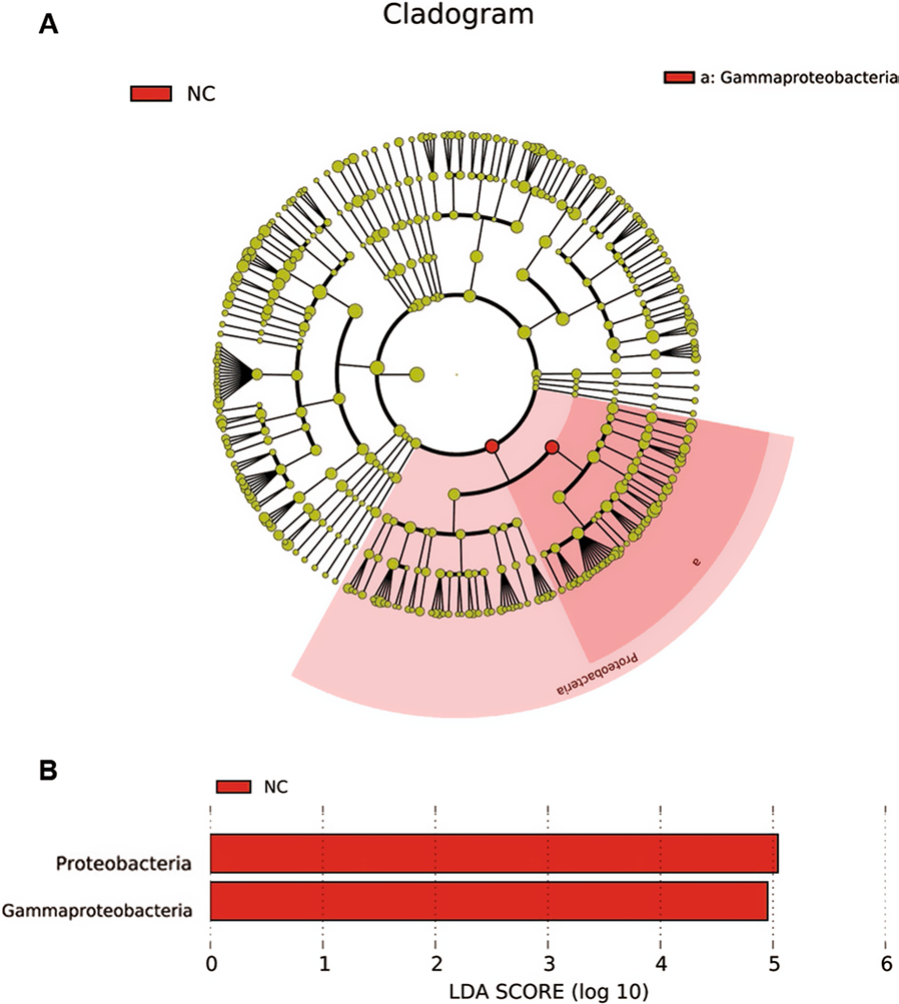
LEfSe analysis of the three groups. **A** A cladogram of the indicative taxa in the vaginal microbiota of the NC group compared to the other two groups. The point in the center represents the root of the tree (bacteria). The rings represent the taxonomic levels in descending order, from phylum to genus. The diameters of the circles represent the relative taxonomic abundances. **B** Histogram of the LDA scores of the taxa enriched in the NC group. The LDA scores indicate the effect sizes and rankings of the taxa with different relative abundance between the NC and P/C groups. (LDA, linear discriminant analysis)

**Fig. 5:**
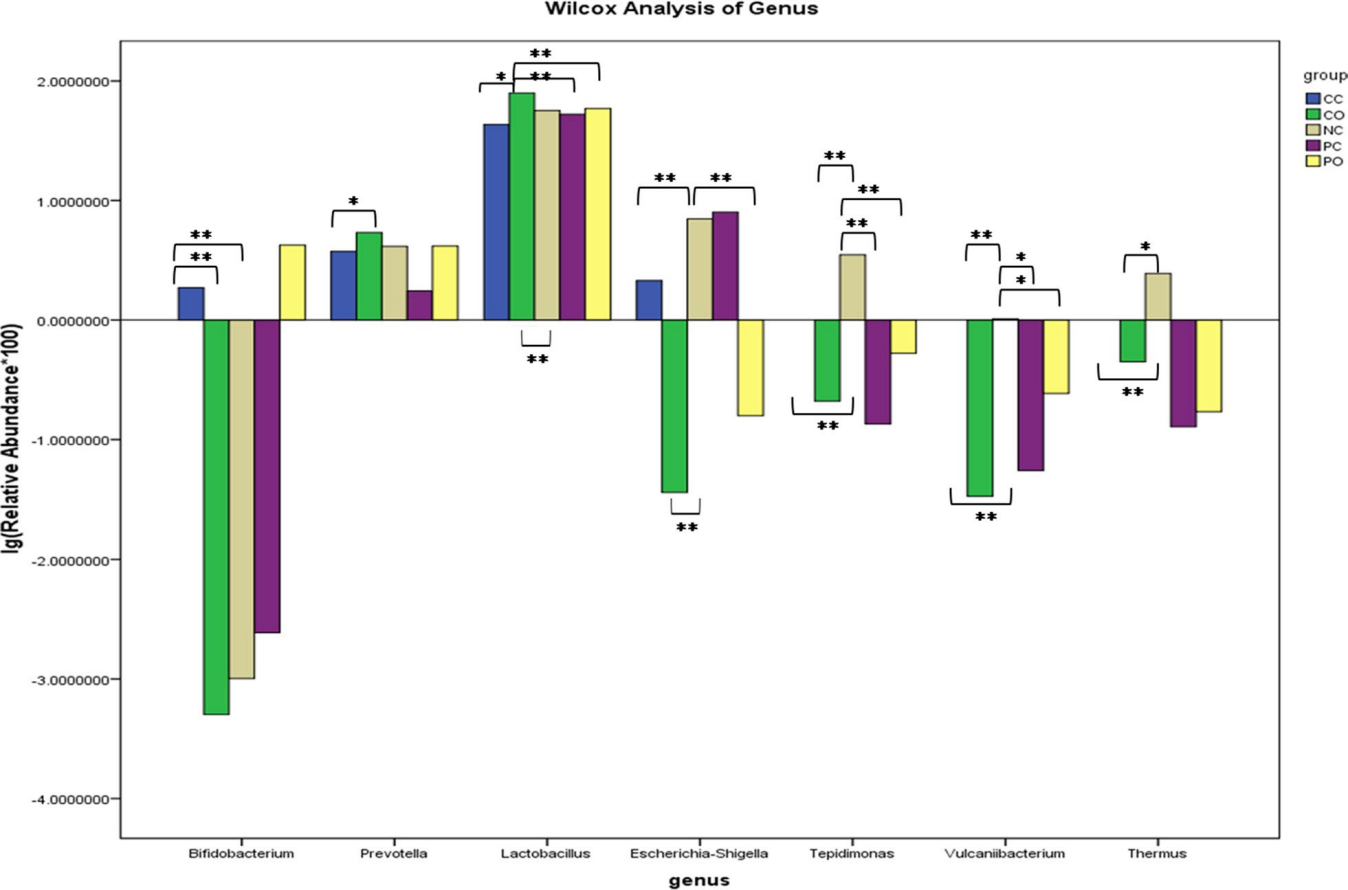
The Wilcox rank-sum test of the NC, PO, PC, CO, and CC subgroups. The horizontal axis indicates different genus, and the vertical axis indicates the mean relative abundance of the genus. The bars in different colors represent different groups. (PC: persistent infection with subtype HPV16 or 18. PO: persistent infection with subtypes other than 16 and18. CC: clearance of subtype16 or18. CO: clearance of subtypes other than 16 and18. *: P < 0.05; **: P < 0.01)

To identify the feature bacterial taxa, the samples of the three groups were compared by LEfSe method. Proteobacteria phylum differed significantly in the mean relative abundance in the NC group, while the P and C groups had no such indicative taxa (Fig. 4).To identify whether there is any biomarker for HPV16 and HPV18 infection or elimination, the subgroups PC (persistent infection with subtype 16 or 18), PO (persistent infection with subtypes other than 16 and 18), CC (clearance of subtype16 or 18), and CO (clearance of subtypes other than 16 and 18) were analyzed further. The Wilcox rank-sum test indicated that genera *Vulcaniibacterium* and *Tepidimonas* of the NC group had a statistically higher mean relative abundance compared to the PO, PC, CO, and CC subgroups (P < 0.05). *Escherichia-Shigella* of the NC group had a higher mean relative abundance compared to the CC, CO, and PO subgroups (P < 0.05). *Thermus* of NC group had a higher mean relative abundance compared to CC and CO groups (P < 0.05). Compared to the CO and NC groups, the *Bifidobacterium* was highly abundant in the CC group (P < 0.05). The *Lactobacillus* in the CO group was in a high mean relative abundance (P < 0.05) compared to the NC, CC, PC and PO groups. Compared to the CC group, *Prevotella* had a higher mean relative abundance in the CO group (P < 0.05) (Fig. 5). Some other genera differed significantly, but the mean relative abundance was very low (< 0.01%), so these genera were ignored.

## Discussion

Accumulating evidence showed that the human microbiota mirrors the host physiology and plays a major role in human health [14]. Some cross-sectional studies have addressed the association between the microbiota in the female reproductive tract and HPV infection and related diseases. Firstly, we attempted to investigate the putative pathogen that might relate to persistent HR-HPV infection without any cytological change through high phylogenetic resolution sequencing and we found that the infection was associated with disturbed microbiota profiles. Although *Lactobacillus* is the most abundant genus in all the three groups, the microbial richness of the NC group was greater than in the C group. The beta diversity analysis revealed a significant difference between the NC and P/C groups. It has been shown that increased diversity of vaginal microbiota has relation with HPV acquisition and persistence [15] However, Gajer et al. [16] found that neither variation in community composition, nor high levels of diversity are necessarily indicative of dysbiosis**.** Our results indicated that P and C groups had the lower vaginal microbiota diversity. It revealed that the alpha and beta diversities of the P and C groups were similar and could be interpreted as part of the samples in C group with infection of more than one subtype HR-HPVs not achieving complete elimination of all the infected HR-HPVs; thus, there was some degree of similarity in the vaginal microbiota of the C and P groups.

The microbial community analysis showed that P and C group samples were associated with an increase in Firmicutes and Actinobacteriota while a decline in Proteobacteria compared to the samples of the NC group. Also, LEfSe analysis found that the most abundant discriminant taxon was Proteobacteria in the NC group. Studies revealed that cervicovaginal dysbiotic states reduce cervicovaginal barrier function [17]and alter metabolic profiles [18], and these may, in turn, facilitate HPV acquisition and CIN/cancer development, respectively. So, the increased abundance of Proteobacteria in vagina may facilitate keeping microbiota stability.

It has been shown that the reduction of genus *Lactobacillus* also has relation with HPV acquisition and persistence. Wei et al. [19] found the abundance of *Lactobacillales* was significantly decreased in the HPV-positive group as compared to the HPV-negative group. Chao et al [20] also found that persistent HR-HPV infection group had a lower relative abundance of *Lactobacillus* than the incident infection and uninfected groups but without statistical difference. In our study, comparing to the NC group, the relative abundance of *Lactobacillus* decreased a little in the P group without statistical difference but increased in the C group significantly. In the subgroup analysis, it revealed that samples of clearing HPV16 or 18 had a higher mean relative abundance of *Bifidobacterium,* and samples of clearing the other HR-HPVs had a higher mean relative abundance of *Lactobacillus* compared to the NC groups. In addition*, Lactobacillus* and *Bifidobacterium* were significantly higher in the CO and CC groups, respectively, indicating that *Bifidobacterium* might exert a protective effect against HPV16 and 18, while *Lactobacillus* might be protective against the other HR-HPVs.

*Bifidobacterium* and *Lactobacillus* have been studied extensively in the virus infection. In an animal experiment, the mice infected with influenza were fed probiotics of *Lactobacillus mucosae* 1025, *Bifidobacterium breve* CCFM1026, and their mixture MIX for 19 days. *B. breve* CCFM1026 significantly increased the lymphocyte count and the expressions of TLR7, MyD88, TRAF6, and TNF-α to restore the immune balance and decrease viral loading [21]. Another study revealed that protein-based metabolic products of *L. casei*, *L. fermentun*, *B. adolescentis*, and *B. bifidum* showed significant anti-rotavirus activity [22]. *B. adolescentis* was also reported as a microorganism with a potential antiviral activity against herpes simplex virus type 1, hepatitis B virus, and coxsackie virus [23–25]. A recent study [26] analyzed the expression of *CASP3* and HPV18 *E6* and *E7* genes in HeLa cells before and after treatment with *L. crispatus* and *L. rhamnosus* culture supernatants. The results indicated that the expression of HPV18 E6 in HeLa cells was significantly decreased after treatment with *Lactobacilli* culture supernatants. These studies suggested that *Lactobacillus* and *Bifidobacterium* were promising anti-HPV infection probiotics but further investigation into the prophylactic properties and mechanisms was needed.

To date, there is no definitive treatment for HPV, however, probiotics may be an effective treatment. Several probiotics comprised of living bacteria (such as *Bifidobacteria* species, *Lactobacilli*, and *Streptococci*), have been proven to improve the immune system and inflammatory state clinically and also reduce the risk of diabetes, allergic disorders, and certain cancers [27]. Palma et al. found that antibiotic treatment plus vaginal *Lactobacillus* implementation was effective in viral clearance in women affected by bacterial vaginosis with concomitant HPV infections [28]. Another clinical trial demonstrated that oral administration of HPV16 E7-expressing *L. casei* resulted in the regression of HPV16-related CIN3. Therapeutic vaccine immunization with E7-bound *L. casei* showed the induction of E7-specific mucosal IFNc-producing cells [29]. Therefore, considering these findings and our results, probiotics containing *Bifidobacteria* and/or *Lactobacillus* may be helpful in HR-HPV clearance in HPV-positive women.

## Conclusions

In conclusion, persistent HR-HPV infection might be associated with dysbiosis of the vaginal microbiota. Microbiome regulation with *Bifidobacteria* and *Lactobacillus* may affect the clearance of HPV, but further research is needed to provide an in-depth insight into the prophylactic properties and the antivirus mechanism.

## Supplementary Information

**Additional file 1.** Rarefaction curves. The horizontal axis indicates the sampling depth, and the vertical axis indicates the index. All the curves become flat as the sampling depth increases, indicating that the high sampling coverage was achieved in all samples.

## Data Availability

The datasets used and analyzed during the current study were deposited into the Genome Sequence Archive (GSA) database. Before released, they are available from the corresponding author on reasonable request.

## Abbreviations

ANOVA: One-way analysis of variance
Anosim: Analysis of similarity
CIN: Cervical intraepithelial neoplasia
CSTs: Community state types
HR-HPV: High-risk human papilloma virus
LDA: Linear discriminant analysis
LefSe: Linear discriminant analysis effect size
NILMs: Negative intraepithelial lesions or malignancies
PcoA: Principal co-ordinate analysis
qRT-PCR: Real-time quantitative polymerase chain reaction
STIs: Sexually transmitted infections
WHO: World Health Organization
