# Supplementary figures and images for: Dysbiosis of vaginal microbiota associated with persistent high-risk human papilloma virus infection
Source: J Transl Med. 2022 Jan 3;20:12. doi: 10.1186/s12967-021-03201-w (PMC8722078; doi:10.1186/s12967-021-03201-w)

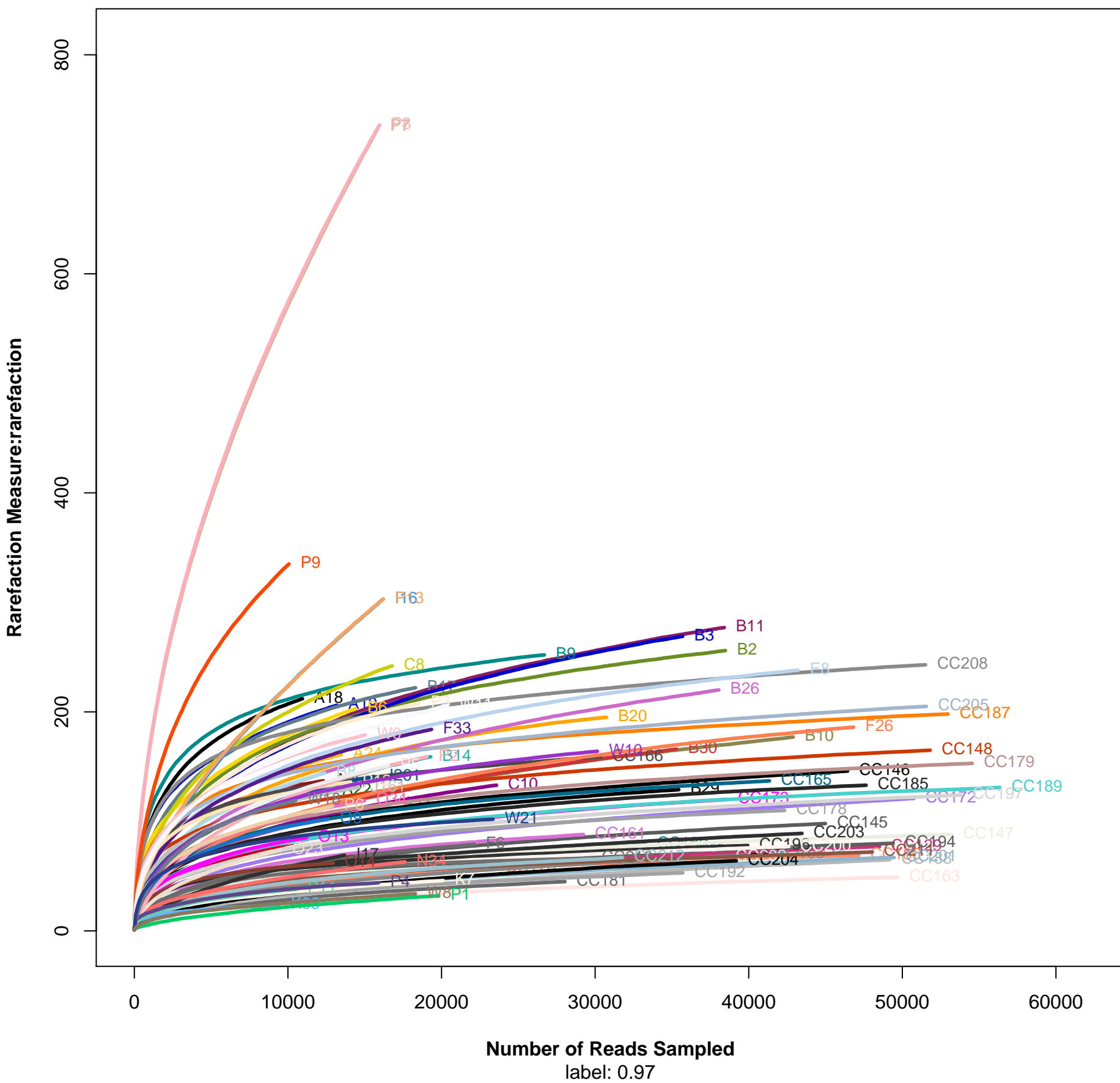

Supplement: Supplementary file 1 — Additional file 1. Rarefaction curves. The horizontal axis indicates the sampling depth, and the vertical axis indicates the index. All the curves become flat as the sampling depth increases, indicating that the high sampling coverage was achieved in all samples. [file 12967_2021_3201_MOESM1_ESM.pdf]
